# Eye‐Tracking Analysis in Surface‐Guided Radiation Therapy Positioning: A Comparative Study of Experienced and Novice Radiation Therapists

**DOI:** 10.1002/jmrs.70044

**Published:** 2025-11-23

**Authors:** Hidetoshi Shimizu, Tomoki Kitagawa, Koji Sasaki, Takahiro Aoyama, Naoki Hayashi, Keisuke Yasui, Takeshi Kodaira

**Affiliations:** ^1^ Department of Radiation Oncology Aichi Cancer Center Hospital Nagoya Aichi Japan; ^2^ Division of Medical Physics, School of Medical Sciences Fujita Health University Toyoake Aichi Japan; ^3^ Graduate School of Radiological Technology Gunma Prefectural College of Health Sciences Maebashi Gunma Japan

**Keywords:** eye tracking, patient positioning, radiation therapist, radiotherapy, SGRT

## Abstract

The patient setup using the surface‐guided radiation therapy (SGRT) system differs from conventional surface marker procedures. Owing to the abundance of three‐dimensional information, there may be operator variability in where to focus during the patient setup. This study aimed to clarify the differences between expert and novice operators in SGRT positioning for head and neck cases by tracking their eye movements, thereby providing data for developing efficient patient setup procedures. Six radiation therapists set up a simulated patient on the SGRT system while recording eye movements on the screen using the QG‐PLUS eye‐tracking system. The positioning time and number of gaze fixations on the screen were analysed, and the relationship between years of experience with SGRT, positioning time and number of gaze fixations was evaluated. No significant correlation was found between SGRT experience and positioning time (*r* = −0.67, *p* = 0.15). However, more experienced radiation therapists exhibited fewer gaze fixations per positioning session (*r* = −0.81, *p* < 0.05), indicating that they efficiently identified key positioning points. Additionally, experienced radiation therapists focused more intently on a specific screen during the latter half of positioning, suggesting a refined approach for final patient alignment verification. More experienced radiation therapists showed fewer gaze fixations and demonstrated increased attention to a specific screen during the latter half of the patient setup process, suggesting that eye‐tracking technology may provide useful data for standardising patient setup procedures in SGRT patient setups.

## Graphical abstract

This study compared expert and novice radiation therapists during surface‐guided radiation therapy (SGRT) setup for head and neck cases using eye tracking. Experts exhibited fewer gaze fixations and concentrated on specific screens during the latter half of positioning, suggesting that they efficiently identified key positioning points. Eye tracking may facilitate the optimization and standardisation of SGRT setup procedures by revealing expert visual strategies.

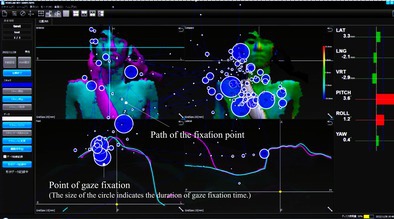

## 1 Introduction

Surface‐guided radiation therapy (SGRT) has become increasingly common, supported by practical guidelines [1, 2]. Most systems provide adequate positional accuracy within recommended tolerances [3, 4, 5, 6, 7], ensuring reliable patient setup. SGRT has proven particularly effective in deep inspiration breath‐hold (DIBH) techniques for breast cancer, enabling partial elimination of skin marks and improved motion management [8].

Unlike conventional surface marker methods, SGRT setup involves abundant three‐dimensional (3D) information, potentially leading to variability among operators. SGRT setup requires training; inexperience may reduce accuracy and prolong setup. These issues are particularly noticeable in highly mobile regions, such as the head and neck.

As a solution to these challenges, we focused on eye‐tracking technology. Eye‐tracking is a method of measuring and tracking where a person is looking and the movement of the eyes relative to the head [9]. Eye‐tracking has been applied in marketing to study gaze‐mediated decision making [10]. In the medical imaging field, a 1963 report by Thomas and Lansdown showed that radiologists' gaze patterns are generally unique to each individual, with a tendency for image coverage to be uneven during reading [11]. In radiation therapy, Kyroudi et al. also analysed the eye movements of radiation oncologists and medical physicists when reviewing treatment planning screens, which allowed us to identify differences in their focus points during the review [12].

This study explores gaze differences between expert and novice radiation therapists using SGRT during head and neck positioning.

## 2 Methods

### 2.1 Surface Image‐Guided Radiation Therapy System

**FIGURE 1:**
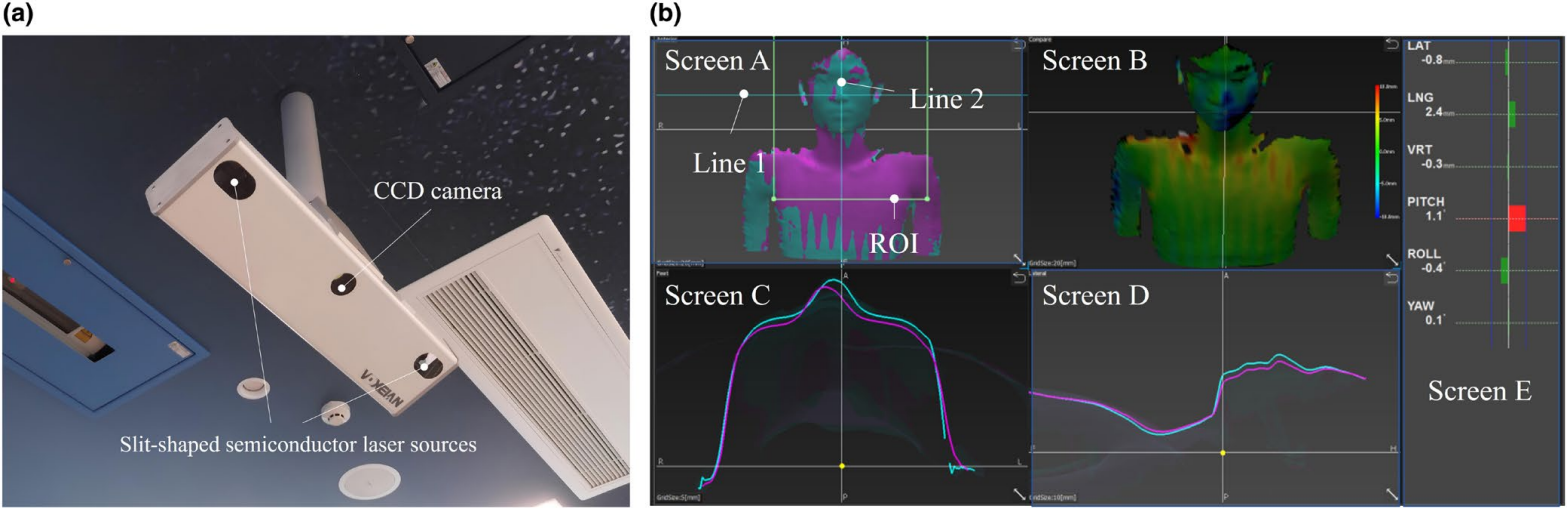
Image (a) shows the VOXELAN system: A 3D measurement device based on the light‐section method, where a slit‐shaped semiconductor laser scans the object, and a charge‐coupled device (CCD) camera captures the reflected light to construct a 3D shape. Image (b) displays the VOXELAN screen. Instructions for viewing each screen are outlined in the text body (refer to the main text for the explanation of screens A to E).

The SGRT device used for patient positioning was the VOXELAN (ERD Co., Okayama, Japan). VOXELAN is a 3D measurement device based on the light‐section method in which a slit‐shaped semiconductor laser scans the object, while a charge‐coupled device (CCD) camera captures the reflected light to construct a 3D shape (Figure 1a). This enables the acquisition of a patient's body position on a couch through non‐contact measurement using the laser, as well as the gathering of comprehensive 3D shape data of the patient's body surface. As shown in Figure S1, the VOXELAN CCD camera is mounted at a ceiling position of −534 mm in the IEC‐Y direction and a linear distance of 1740 mm from the virtual isocenter of a radiation treatment machine (Radixact; Accuray Inc., Sunnyvale, CA).

As shown in Figure 1b, the position of the patient on the couch can be evaluated in real time by acquiring a reference image in advance. Screen A displays the reference image superimposed on the real‐time image. The reference image is light blue, whereas the real‐time image is purple. The areas where purple appears indicate that the real‐time image is at a higher position (closer to the CCD camera) than the reference image (light blue indicates the opposite). Screen B illustrates the positional difference between the reference and real‐time images, with red‐to‐blue contrast showing a shift from 10 to −10 mm. Red indicates that the real‐time image is at a higher position than the reference, whereas blue indicates that the real‐time image is at a lower position. Green indicates good agreement. Screen C presents the cross‐sectional profile image of line 1 from screen A. The light blue line represents the reference image profile and the purple line represents the real‐time image profile. Similarly, screen D shows the cross‐sectional profile at line 2 from screen A. Finally, screen E displays the displacement in each of the six axes (three axes of translation: lateral, longitudinal and vertical, and three axes of rotation: pitch, roll and yaw) between the reference image and the real‐time image in the region of interest (ROI) of screen A. The positional accuracy specification for the misalignment of the VOXELAN was within 1 mm and 1° [4].

### 2.2 Eye‐Tracking System

**FIGURE 2:**
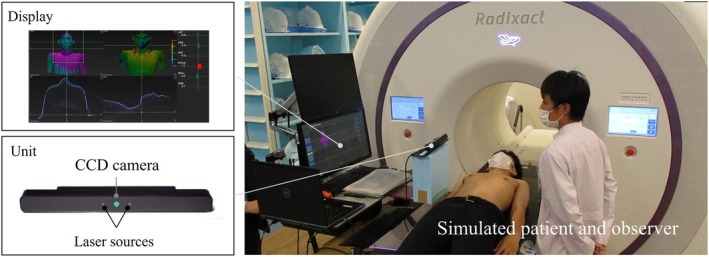
Arrangement of each system for positioning a simulated patient: The QG‐PLUS eye‐tracking system (bottom left) and the VOXELAN (top left).

The QG‐PLUS eye‐tracking system (DITECT Co. Ltd., Tokyo) was used [13]. This system emits near‐infrared light from the LEDs mounted on both sides of the camera in the unit (bottom left of Figure 2). It utilises corneal reflection to locate the pupil without requiring specialised goggles. Calibration for eye‐gaze position involved a simple interface, completed by gazing at the marker points displayed on the screen in sequence. The optimal measurement distance was 40–75 cm, with a head movement range of 31.5 cm horizontally, 22.5 cm vertically, and 35 cm forward and backward relative to the unit. The accuracy was 0.5° and the measurement cycle was 60 fps. The QG‐PLUS eye‐tracking system provides functions to analyse both the number of times and the duration in which the gaze is fixed on each area of the screen.

### 2.3 Eye‐Tracking Procedure

A simulated patient used a pillow (Silverman Headrest, B type; CQ MEDICAL), which is widely employed in radiation therapy for patients with head and neck cancer, and lay on the couch to obtain the reference image of the SGRT image. The simulated patient was then asked to get off the couch once, and when the simulated patient returned to the couch, the couch was adjusted to a position near the reference image. In this state, positioning was initiated by a radiation therapist (RT). During positioning, eye‐tracking analysis was performed, as shown in Figure 2. The RT adjusted the simulated patient using a combination of methods, including couch translational movements, manual adjustments of the simulated patient's body, and verbal instructions while monitoring the VOXELAN screen. Once the positioning accuracy was within acceptable limits (5 mm and 1°), the RT informed the subject that the process was complete and the eye‐tracking analysis was terminated. Six radiation therapists performed the procedure. Furthermore, we aimed to keep the simulated patient's and couch's positions as consistent as possible at the start of each eye‐tracking session. Table 1 shows the years of positioning experience of each RT with the SGRT system. All radiation therapists have more than five years of experience. Radiation therapists number 2–5 had received several weeks of on‐the‐job training in SGRT positioning as part of their routine clinical duties under the supervision of RT No. 6. One individual without any prior SGRT experience was given a brief explanation of how to interpret the VOXELAN screen before participating in the experiment.

**TABLE 1:** Each radiation therapist's years of positioning experience with the SGRT system.

Radiation therapist No.	Years of positioning experience with the SGRT system [years]
1	0
2	0.5
3	1
4	1
5	1.5
6	4

This study was approved by the institutional review boards of Fujita Health University (EN25‐015) and Aichi Cancer Center (IR061134).

## 3 Data Analysis

Figure S2 presents an example of eye‐tracking analysis conducted on the VOXELAN screens. The blue circle marks the fixation point, and the size of the circle represents the fixation duration. The blue lines connecting the visible circles on the body indicate the path of the fixation point. Based on these data, the positioning time and number of gaze fixations on the VOXELAN screen (/minute) were analysed for six radiation therapists. Fixation was defined as gaze pauses of 10 ms or longer, indicating that up to 6000 fixations per minute may occur, and the relationship between years of SGRT positioning experience and positioning time or the number of gaze fixations (/minute) was assessed using Spearman's rank correlation coefficient. All reported *p* values were based on two‐tailed tests, with the significance level set at *p* < 0.05. All statistical analyses were performed using EZR [14], a modified version of R Commander designed to include statistical functions commonly used in biostatistics.

## 4 Results

### 4.1 Relationship Between Years of Positioning Experience With the SGRT System and Positioning Time

**FIGURE 3:**
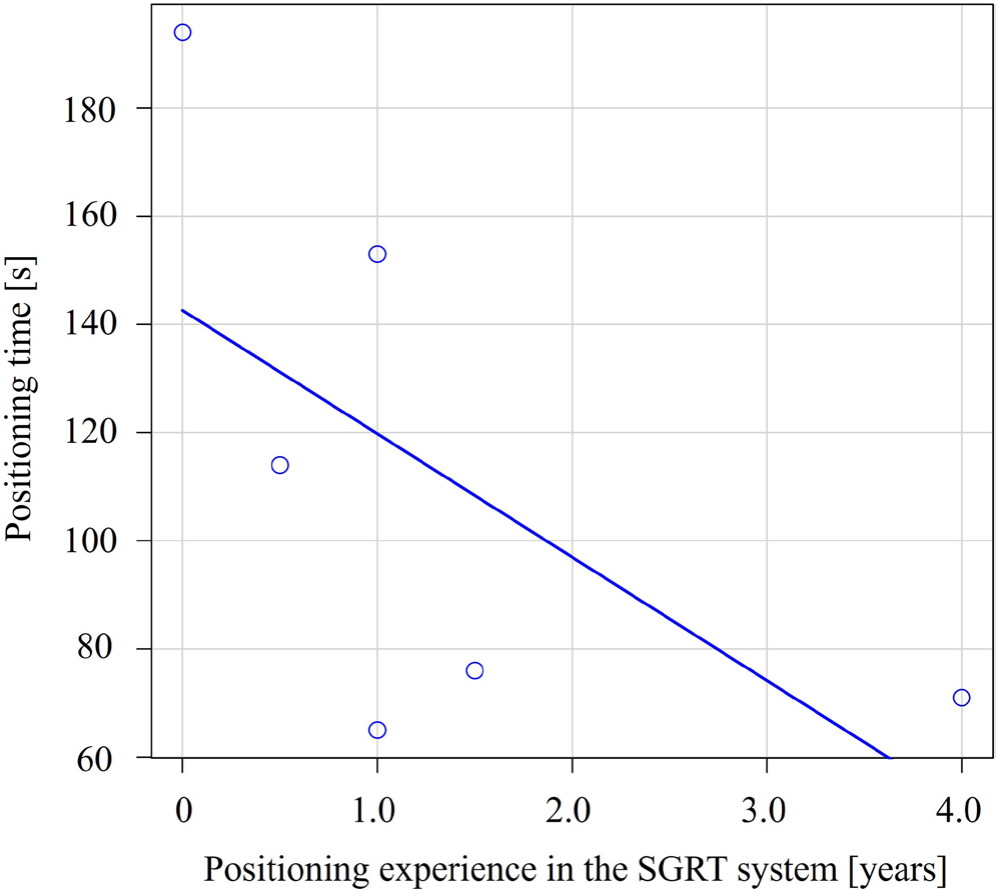
The relationship between years of positioning experience with the SGRT system and positioning time (*r* = −0.67, *p* = 0.15).

Figure 3 shows the relationship between years of positioning experience with the SGRT system and positioning time; there was no significant correlation between them (*r* = −0.67, *p* = 0.15).

**FIGURE 4:**
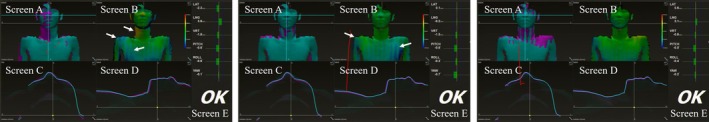
The final setup screens of the VOXELAN of the radiation therapists (left: RT No. 1, middle: RT No. 5, right: RT No. 6; see Table 1 for years of positioning experience with the SGRT system). Arrows in screen B indicate areas of greater discrepancy between the real‐time and reference images. Red indicates that the real‐time image is at a higher position than the reference, while blue indicates that the real‐time image is at a lower position.

Figure 4 shows the final VOXELAN screens from radiation therapists No. 1, 5 and 6 (see Table 1 for experience years). Reference and real‐time image profiles on screens C and D matched well, but discrepancies were noted on screen B. In RT No. 1, red appeared around the right neck, and blue around the right shoulder and chest. RT No. 5 showed similar blue areas on the right shoulder and left chest. In contrast, RT No. 6 displayed only slight blue on the right shoulder, with the rest mostly green, indicating better alignment. Although setups were based on individual judgment, differences in final patient positions were evident.

### 4.2 Relationship Between Years of Positioning Experience With the SGRT System and the Number of Gaze Fixations

**FIGURE 5:**
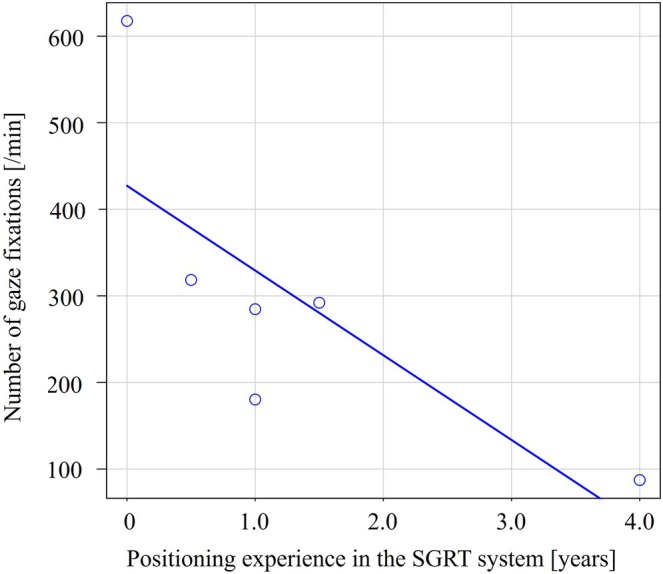
The relationship between years of positioning experience with the SGRT system and the number of gaze fixations (/minute); a negative correlation was observed (*r* = −0.81, *p* < 0.05).

Figure 5 shows the relationship between years of positioning experience with the SGRT system and the number of gaze fixations (/minute); a negative correlation was observed, indicating that the number of gaze fixations tended to decrease as experience with the SGRT system increased (*r* = −0.81, *p* < 0.05).

**FIGURE 6:**
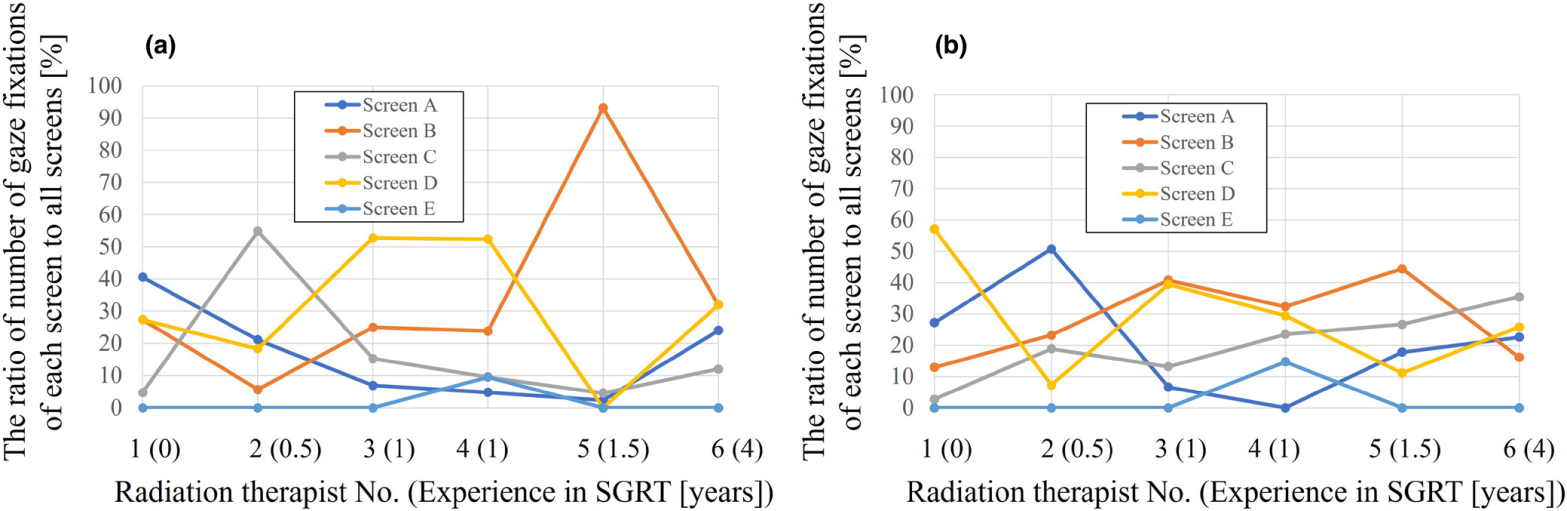
The percentage of gaze fixations on each screen, dividing each radiation therapist's positioning time into the first and second halves (a and b, respectively). In the second half, screen C showed a positive correlation (*r* = 0.97, *p* < 0.05) with years of positioning experience with the SGRT system.

Figure 6 shows gaze fixation percentages by screen, split into the first and second halves of positioning time. In the first half, some radiation therapists focused on specific screens (e.g., screen C for RT No. 2, screen B for RT No. 5), while in the second half, their gaze became more evenly distributed. Fixation ratio on screen C positively correlated with SGRT experience (*r* = 0.97, *p* < 0.05). The radiation therapists with greater SGRT experience appeared to spend a larger proportion of their fixation ratio on screen B, although this trend was not statistically significant (first half: *r* = 0.38, *p* = 0.45; second half: *r* = 0.27, *p* = 0.61). Screen E was used infrequently throughout. Videos S1 and S2 show the eye‐tracking for radiation therapists No. 1 and 6.

## 5 Discussion

This study investigated gaze differences between expert and novice radiation therapists during head and neck positioning with SGRT. Although positioning time was not significantly associated with experience, more experienced radiation therapists showed fewer gaze fixations. Additionally, experience was positively correlated with increased attention to a specific screen.

Krupinski et al. used virtual slide technology to compare the eye movements of virtual slide readers with different experience levels. They found that pathologists spent significantly less time viewing each slide image than residents or medical students [15]. In addition, experienced radiologists in mammography are known to spend significantly less time making decisions per case [16]. Our study found no significant correlation between years of SGRT experience and positioning time. This discrepancy is likely due to differences in the accuracy of the final patient positioning. Although all radiation therapists achieved positioning within the acceptable limits (5 mm and 1°), variations were observed in the final position of the simulated patient (Figure 4). Notably, the results from the RT with the most positioning experience with the SGRT system showed good agreement between the reference and real‐time images, whereas setups from other radiation therapists exhibited slight deviations (Figure 4). These findings indicate that proficiency with the SGRT system takes time and that setup accuracy may vary depending on the radiation therapist's experience. Standardising SGRT positioning goals and correction methods is essential.

In chest radiography, experienced radiologists are known to make fewer saccades (rapid eye movements) than less experienced radiologists [17]. Similarly, we found that SGRT experience correlated with fewer gaze fixations per minute (*r* = −0.81, *p* < 0.05). This suggests that less experienced radiation therapists struggle to identify efficient positioning points, while experienced ones focus more clearly. Furthermore, in the second half of the positioning, only screen C showed a positive correlation with SGRT experience (*r* = 0.97, *p* < 0.05), suggesting its role in final adjustments. However, it should be noted that a lower number of gaze fixations or focusing on specific screen areas does not necessarily equate to higher positioning accuracy. What is important to note is that, as shown in Videos S1 and S2, eye‐tracking technology allows us to understand what misalignment adjustments were made and at what timing during patient positioning, as well as the specific approach taken. Sharing this information could clarify the appropriate procedures and objectives, thereby improving the positioning accuracy with the SGRT system. Eye‐tracking technology is expected to provide unprecedented learning information and contribute to the standardisation of positioning with the SGRT system, which contains a large amount of data. Furthermore, by sharing this gaze analysis data among radiation therapists and establishing standardised patient setup procedures in SGRT, it is possible to advance further research, such as investigating whether applying these procedures can accelerate the learning curve for novice radiation therapists.

This study had some limitations. Six radiation therapists were surveyed for a simulated patient; however, the limited sample size number of repetitions make it unclear whether the results can be generalised to other radiation therapists. Validation with a larger number of simulated patients and radiation therapists could increase the reliability of the results. In addition, data on the learning effects of eye‐tracking are lacking and remain a topic for future research. Next, the ROI was defined as shown in Figure 1 and kept fixed across simulated patient positioning and among radiation therapists. In this study, a relatively broad ROI was employed; however, due to the high anatomical mobility of the head and neck, this definition might have limited the reliability of screen E.

## 6 Conclusions

This study investigated differences in gaze behaviour between experienced and novice radiation therapists during head and neck positioning using the SGRT system. More experienced radiation therapists showed fewer gaze fixations and demonstrated increased attention to a specific screen during the latter half of the patient setup process. Further studies with larger cohorts are needed to validate and generalise these results. Eye‐tracking may support standardisation of patient setup procedures and improve positioning consistency in SGRT.

## Author Contributions

Hidetoshi Shimizu conceived the study, analysed and interpreted the data, and drafted the manuscript. Tomoki Kitagawa, Koji Sasaki, Takahiro Aoyama, Naoki Hayashi, Keisuke Yasui and Takeshi Kodaira participated in the study design and made significant contributions to editing the manuscript. All authors have read and approved the final manuscript.

## Funding

This work was supported by the JSPS KAKENHI Grant Numbers 21K15817 and 24K18847.

## Ethics Statement

This study was approved by the institutional review boards of Fujita Health University (EN25‐015) and Aichi Cancer Center (IR061134).

## Conflicts of Interest

The authors declare no conflicts of interest.

## Supporting information


**Figure S1:** The VOXELAN CCD camera is positioned at a ceiling height of −534 mm in the IEC‐Y direction and 1740 mm in linear distance from the virtual isocenter of a radiation treatment machine (Radixact; Accuray Inc., Sunnyvale, CA). The elevation angle between the couch and the camera at the virtual isocenter position is 74°.

**Figure S2:** An example of eye‐tracking analysis on the VOXELAN screens. The blue circle indicates the point of gaze fixation, and the size of the circle indicates the duration of gaze fixation time. Additionally, the blue lines connecting the circles visible on the surface of the body illustrate the path of the fixation point.

**Video S1:** Eye tracking for radiation therapist No. 1.

**Video S2:** Eye tracking for radiation therapist No. 6.

## Data Availability

The data that support the findings of this study are available from the corresponding author upon reasonable request.
