# Supplementary figures and images for: Eye‐Tracking Analysis in Surface‐Guided Radiation Therapy Positioning: A Comparative Study of Experienced and Novice Radiation Therapists
Source: J Med Radiat Sci. 2025 Nov 23;73(1):115–20. doi: 10.1002/jmrs.70044 (PMC12950502; doi:10.1002/jmrs.70044)

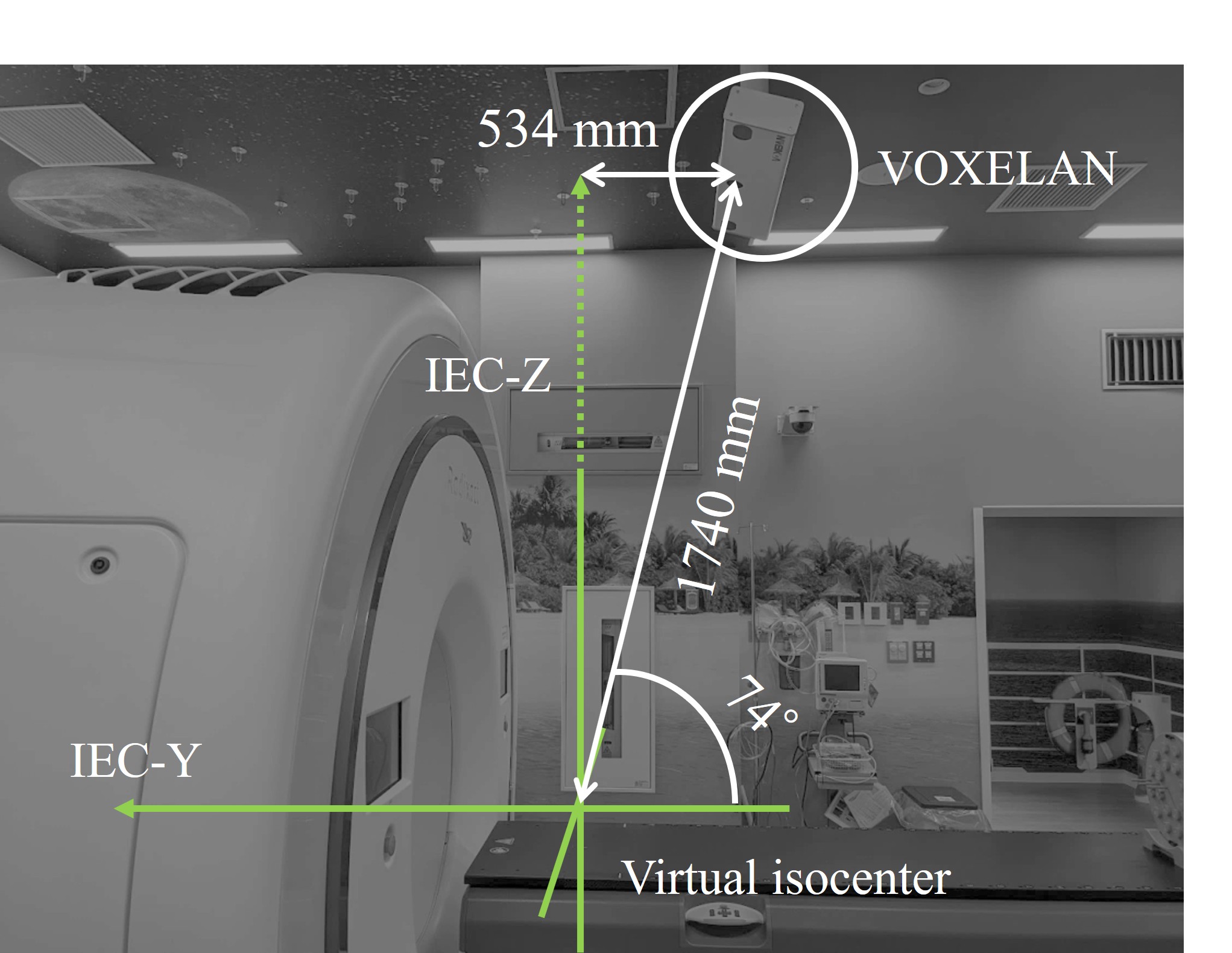

Supplement: Supplementary file 1 — Figure S1: The VOXELAN CCD camera is positioned at a ceiling height of −534 mm in the IEC‐Y direction and 1740 mm in linear distance from the virtual isocenter of a radiation treatment machine (Radixact; Accuray Inc., Sunnyvale, CA). The elevation angle between the couch and the camera at the virtual isocenter position is 74°. [file JMRS-73-115-s004.jpg]

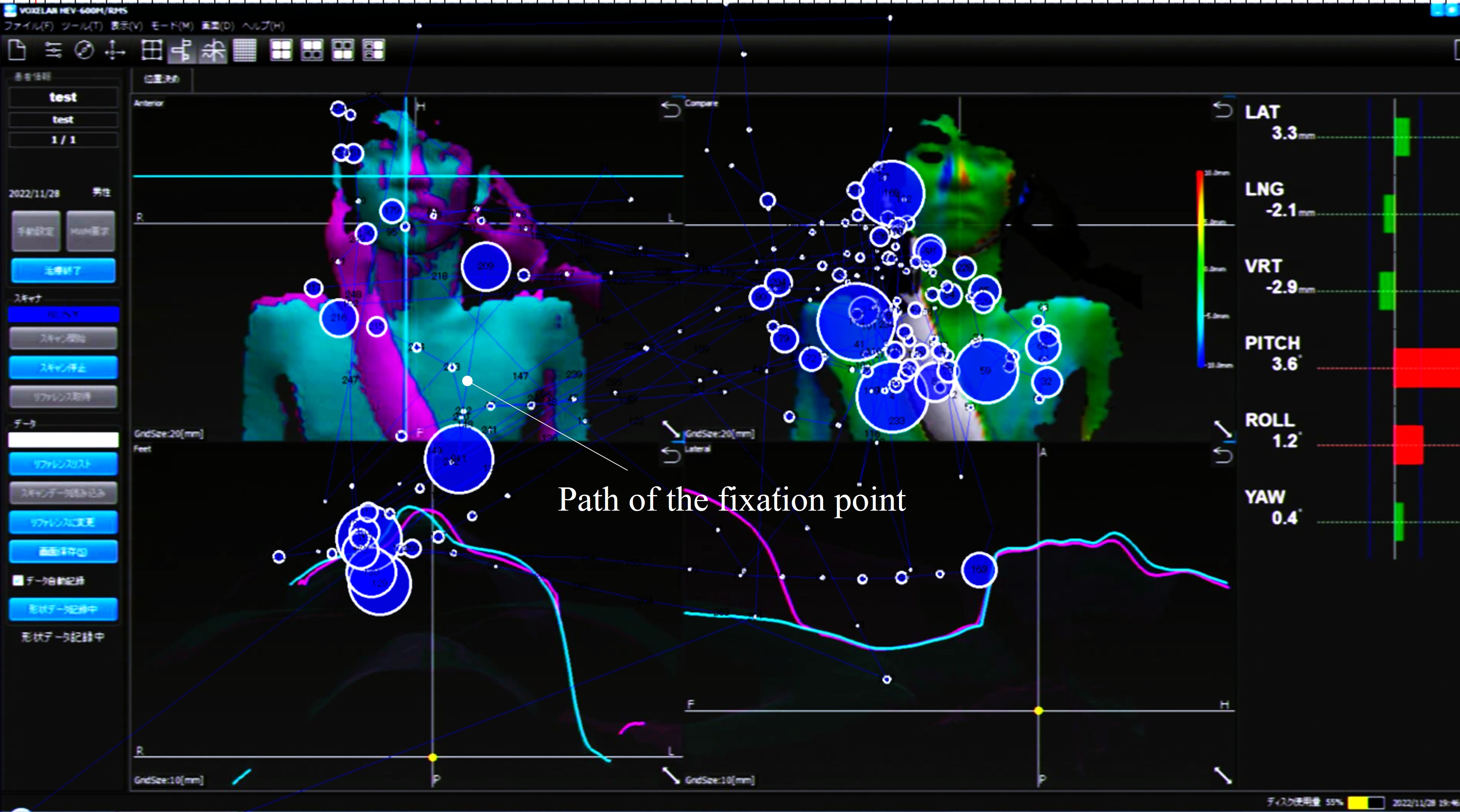

Supplement: Supplementary file 2 — Figure S2: An example of eye‐tracking analysis on the VOXELAN screens. The blue circle indicates the point of gaze fixation, and the size of the circle indicates the duration of gaze fixation time. Additionally, the blue lines connecting the circles visible on the surface of the body illustrate the path of the fixation point. [file JMRS-73-115-s001.jpg]
